# Effectiveness of Moderate Intensity Interval Training as an Index of Autonomic Nervous Activity

**DOI:** 10.1155/2016/6209671

**Published:** 2016-11-10

**Authors:** Satoru Kai, Koji Nagino, Takayoshi Ito, Rie Oi, Kazushi Nishimura, Shuhei Morita, Riyo Yaoi

**Affiliations:** Division of Physical Therapy, Department of Rehabilitation Sciences, Faculty of Allied Health Sciences, Kansai University of Welfare Sciences, 3-11-1 Asahigaoka, Kashiwara City, Osaka 582-0026, Japan

## Abstract

The purpose of this study was to examine the effects of moderate intensity interval training from the change of the autonomic nervous activity. Ten male volunteers aged 21-22 years were studied. After 10-minute rest in a seated position, the subjects were asked to perform the strength of moderate cycling exercise in ergometer. Cycling rate was done in 50 times/min. Load resistance of the ergometer was set to 2.0 kgm. Subjects paused the exercise when the heart rate becomes 120 beats/min. Subjects have resumed the exercise when the heart rate returns to the value at rest. This trial was repeated twice. The experiment was ended when the heart rate of the subjects has returned to resting level. When the heart rate during exercise is maintained to less than 120 beats/min, sympathetic nerve activity during exercise did not work actively compared to the baseline. Vagus nerve activity after exercise cessation exceeds the baseline. It is clarified that the exercise as well as activating the vagus nerve activity stimulates the total autonomic nervous activity. It has revealed that at the time of interval training at moderate load the vagus nerve activity can be carried out.

## 1. Introduction

Exercise is associated with increased sympathetic tone and decreased cardiac vagal nerve activity, leading to decreased heart rate variability [1–4]. These results suggest that endurance training is to activate the vagus nerve activity [5]. Those who are doing the exercise have a significantly higher vagal activity as compared to those who do not [6–8]. Vagus nerve activity by endurance training increases significantly [9, 10]. Interval training is likely to encourage the activation of the vagus nerve activity compared to endurance training in theory.

Autonomic nervous activity decreases with age. Decreasing vagus nerve activity is likely to become heart disease [11]. Sympathetic nerve activity is turned to be dominant; it shows the imbalance of the autonomic nervous activity. Relationship of fatigue and sympathetic nerve activity has been pointed out. As a result, there is a potential to cause heart disease. As a method for improving, exercise has been reported. Heart rate at rest is reduced by the continuation of the exercise [6] and by endurance training [5, 9, 12–14]. For keeping people healthy, it is necessary to find an exercise to suppress the sympathetic activity and to increase the autonomic nervous activity especially vagus nerve activity. Exercise as an index of the heart rate, aerobic exercise, is used in many studies; it shows the change of the autonomic nerve activity. However, the results are often not reached until enhancing vagal activity. Then, we demonstrate the effectiveness of this training from the change of the autonomic nervous activity by moderate intensity interval training in an index of the heart rate.

## 2. Material and Methods

Ten male volunteers aged 21-22 years (height 164.8 ± 10.6 cm, body mass 60.4 ± 13.6 kg, and BMI 22.4 ± 1.6 kg m^−2^) participated in this study. According to the results of questionnaire and electrocardiogram, all subjects were free of hypertension, hyperlipemia, cardiovascular disease, and diabetes mellitus. In addition, they were nonsmokers, and none of them was taking any medicine known to affect cardiovascular function. Subjects were not taking prescribed medications and presented with normal levels of blood pressure and ECG patterns. The study conformed to the recommendations of the Declaration of Helsinki. The study was approved by the Ethics Committee of the Kansai University of Welfare Sciences. All subjects provided written informed consent for their participation in the experimental procedures.

All measurements were performed in a quiet and air-conditioned (22°C) room. All subjects did not consume any beverages containing alcohol or coffee before the measurements. Subjects were allowed to rest comfortably in a seated position on the cycle ergometer. After 10-min quiet rest in a seated position, subjects were asked to perform moderate intensity cycling exercise on an electrically ergometer. The cycling speed was performed at 50 rounds per minutes. Load resistance of the ergometer was set to 2.0 kgm. Subjects were instructed to breathe with expiration adjusted to 4 seconds and inspiration adjusted to 2 seconds. Subjects began the exercise and paused the exercise when the heart rate becomes 120 beats per minute. Subjects have resumed the exercise when the heart rate returns to the value at rest. This trial was repeated twice. Thereafter, the experiment was terminated when the heart rate of the subjects has returned to resting level. From the pre- to postexercise, room temperature and humidity had been kept mostly to, respectively, 22°C and 60%.

The electrocardiogram (ECG) was monitored by Binary light recorder (GMS Companies) during the experiment for heart rate variability (HRV) analysis. Power spectra obtained from spectral analysis were defined as two components: 0.04~0.15 Hz (low frequency: LF) and 0.15~0.4 Hz (high frequency: HF). HF power was shown to be almost entirely mediated by the vagal nerve activity [15], whereas LF power reflects the mixed modulation of vagal and sympathetic nerve activities [16]. The ratio of LF power to HF power (LF/HF) was considered to reflect the sympathovagal balance, and high values suggested sympathetic predominance [17].

According to the previous studies [14, 18, 19], the HF power, LF power, LF/HF ratio, and total power (LF power plus HF power) were transformed into their natural logarithms (ln) before statistical analysis. The logarithmic transformations produced approximately symmetric distributions and thus allowed for the use of parametric statistics that require near normal distribution. Time of analysis each time was to be described as follows: at rest (baseline), during the first round of exercise, at 20–30 seconds after the first round of exercise cessation, at 50–60 seconds after the first round of exercise cessation, during the second round of exercise, at 20–30 seconds after the second round of exercise cessation, at 50–60 seconds after the second round of exercise cessation, during the third round of exercise, at 20–30 seconds after the third round of exercise cessation, at 50–60 seconds after the third round of exercise cessation.

All corresponding data among each period were compared by multiple comparisons. All data were expressed as mean (±SD). Statistical significance was set at *p* < 0.05. Statistical analyses were done using SPSS 22.0 for Windows (SPSS Inc.).

## 3. Results

**Figure 1:**
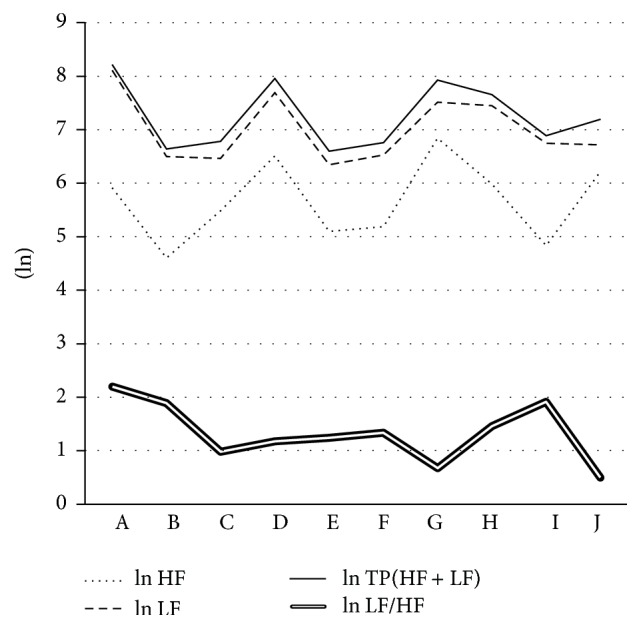
Changes in the autonomic nervous activity of each period. A: baseline (at rest). B: 1st: during exercise (10 sec). C: 1st: at 20–30 sec after exercise cessation (10 sec period). D: 1st: at 50–60 sec after exercise cessation (10 sec period). E: 2nd: during exercise (10 sec). F: 2nd: at 20–30 sec after exercise cessation (10 sec period). G: 2nd: at 50–60 sec after exercise cessation (10 sec period). H: 3rd: during exercise (10 sec). I: 3rd: at 20–30 sec after exercise cessation (10 sec period). J: 3rd: at 50–60 sec after exercise cessation (10 sec period).

Results of heart rate of each period are presented in Table 1. Changes in the autonomic nervous activity of each period are presented in Figure 1. The change in autonomic nervous activity, ln⁡HF and ln⁡LF, was not significantly different at each period. ln⁡TP was significantly different between baseline (at rest) and 1st: during exercise (10 sec), 1st: at 20–30 sec after exercise cessation (10 sec period), 2nd: during exercise (10 sec), and 3rd: at 20–30 sec after exercise cessation (10 sec period) and between 1st: during exercise (10 sec) and 1st: at 50–60 sec after exercise cessation (10 sec period).

## 4. Discussion

Vagus nerve activity decreases immediately after the exercise and increases after the exercise cessation. Increase of the vagus nerve activity after the exercise cessation is delayed at the time of a strong exercise intensity. In this study, the effect of the exercise continuation is not clear, and moderate intensity of exercise is one of the means to increase the vagus nerve activity in order to increase the vagus nerve activity after exercise cessation as immediate change.

When the exercise intensity is gradually increased, the autonomic nervous activity is transferred to dominant sympathetic nerve activity from dominant vagus nerve activity [3, 20]. For keeping people healthy, it is necessary to set the exercise to increase the total autonomic nervous activity and vagus nerve activity and to suppress the sympathetic nerve activity. In the results of this study, when the heart rate during exercise is maintained below 120 beats/min, sympathetic nerve activity during exercise did not work actively compared to the baseline. Vagus nerve activity after exercise cessation exceeds the baseline. It is clarified that the exercise as well as activating the vagus nerve activity stimulates the total autonomic nervous activity. In the previous studies [19, 21, 22], vagus nerve activity after exercise cessation was less to exceed the baseline. It is assumed that load was strong to activate the vagus nerve activity. Current of heart disease rehabilitation is not possible to activate the vagus nerve activity [23], leaving a challenge to the setting of the program.

The reason for using the interval training is an application of the role of the vagus nerve activity. Vagus nerve activity is relaxed to start the exercise, increasing the heart rate. As the exercise load becomes stronger, vagus nerve activity is decreased and sympathetic nerve activity is increased. Sympathetic nerve activity is diminished after exercise cessation. Vagus nerve activity after exercise cessation is enhanced in order to return to the value of the heart rate at rest. From these phenomena, we thought interval training is likely to prompt the activation of the vagus nerve activity compared to the endurance training. The one-time exercise in the load amount of moderate hard to encourage the activation of the vagus nerve activity [19, 22]. By prolonged exercise, it is possible to increase the vagus nerve activity [24]; it should be noted on the kind of exercise. On interval training in this load, it is possible to increase the vagus nerve activity without increasing the sympathetic nerve activity. It was suggested that the interval training to repeat the exercise cessation is effective as a way of stimulation to the heart autonomic nervous activity for activating the vagus nerve activity.

Exercise improves baroreflex function and decreases oxidative stress in cardiovascular diseases linked to elevated central Angiotensin II [25]. Exercise improves abnormal cardiovascular reflexes in chronic heart failure [26]. Exercise is likely to improve the central and peripheral mechanisms of the sympathetic nervous system.

Vagus nerve system has led to worsening of the condition attenuation of the effects of heart failure; vagus nerve stimulation has been attracting attention as an effective new treatment. There is a need to activate the vagus nerve activity by using the exercise for keeping people healthy. There is a relationship between increasing vagus nerve activity after exercise cessation and the value at rest [27]; it is a need to activate the vagus nerve activity. In order to activate the vagus nerve activity, reactions are required in excess of the value at rest. However, in many studies, the increasing vagus nerve activity does not exceed the value at rest level. Therefore, we analyzed the autonomic nervous activity at the time of interval training at moderate intensity. It revealed an increase in the vagus nerve activity after exercise cessation.

## Figures and Tables

**Table 1:** Changes in the heart rate of each period (*n* = 10).

	Mean (beats/min)	Standard deviation
Baseline (at rest)	78.8	9.2
1st: during exercise (10 sec)	115.0	6.4
1st: at 20–30 sec after exercise cessation (10 sec period)	96.3	9.2
1st: at 50–60 sec after exercise cessation (10 sec period)	81.7	9.4
2nd: during exercise (10 sec)	115.0	2.0
2nd: at 20–30 sec after exercise cessation (10 sec period)	96.6	9.0
2nd: at 50–60 sec after exercise cessation (10 sec period)	84.5	9.1
3rd: during exercise (10 sec)	113.9	3.6
3rd: at 20–30 sec after exercise cessation (10 sec period)	98.5	12.4
3rd: at 50–60 sec after exercise cessation (10 sec period)	82.4	8.3
